# Oral Health Impact Profile (OHIP) as a Tool for the Assessment of the Oral Health-Related Quality of Life—A Scoping Review

**DOI:** 10.3390/dj13110490

**Published:** 2025-10-23

**Authors:** Łukasz Wojszko, Karolina Banaszek, Oliwia Gagacka, Joanna Bagińska

**Affiliations:** 1 Department of Dentistry Propaedeutics, Medical University of Bialystok, 15-295 Bialystok, Poland; lukasz.wojszko@umb.edu.pl; 2 Students Scientific Club ‘StuDentio’, Department of Dentistry Propaedeutics, Medical University of Bialystok, 15-089 Bialystok, Poland; 43912@student.umb.edu.pl (K.B.); 43914@student.umb.edu.pl (O.G.)

**Keywords:** oral health-related quality of life, Oral Health Impact Profile, OHIP, adults

## Abstract

**Background/Objectives**: The Oral Health Impact Profile (OHIP) is the most widely used tool for OHRQoL assessment. The measure has several versions, but there is no comprehensive summary of available Oral Health Impact Profile variants. The purpose of this scoping review is to identify and summarize Oral Health Impact Profile versions for the adult population available in the literature. **Methods**: PubMed, Scopus, and Web of Science databases were searched on 25–28 May 2025 to find papers presenting the Oral Health Impact Profile versions’ development process. Records written in English without any time restrictions were included. The Joanna Briggs Institute framework for scoping reviews was applied. The PRISMA-ScR approach was followed. **Results**: In total, 11 generic OHIP scales (the OHIP version that was not targeted at any specific condition) and 16 condition-specified OHIP scales were found. The analysis revealed a wide variety of number of items (from 49 to 5), recall period (from one year to one week), rating scale (4-0; 5-0; 5-1; 6-1; 1, 0, and −1), dimensionality of scale (7, 4, or 3 dimensions, 2–6 factors, or unidimensional), and validation process. **Conclusions**: Differences in OHIP features have to be taken into account during a comparison of results from different studies. Due to the availability of various tools, the idea of creating new versions of the OHIP should be considered with caution. Researchers should carefully select the appropriate OHIP version for their purposes, as the process of adapting the tool to a new language and culture is time-consuming and expensive.

## 1. Introduction

Oral health is undeniably a key component of overall health. As early as the mid-20th century, the World Health Organization (WHO) defined health as not only the absence of disease, but also full physical, mental, and social well-being [1]. In response to this definition, medicine began to take into account the subjective feelings of patients in the treatment process. However, these concepts were not easily introduced into dentistry, and it was not until the 1980s that the importance of the impact of oral diseases on a patient’s overall well-being started to receive greater consideration [2,3]. In contrast, modern dentistry not only focuses on the treatment but also highlights the impact of oral health on an individual’s overall condition and functioning on both the physical and emotional levels. Chronic dental issues such as pain, tooth loss, biting difficulties, aesthetic defects, or facial deformities can significantly reduce life comfort and satisfaction. Measuring oral health-related quality of life (OHRQoL) is an important part of a holistic approach to dental care, helping us to understand how oral diseases affect patients’ general well-being, as well as social, professional, and interpersonal activities [3]. OHRQoL is a key way to obtain dental patient-related outcomes (dPRO) [4,5].

OHRQoL measurement has a wide range of applications—from individual patient assessment in dental practice to studies evaluating the effectiveness of different treatment methods [3,6,7,8,9]. In a clinical setting, OHRQoL allows us to identify a patient’s hidden problems, such as psychological and social issues that may be overlooked by dentists if not specifically examined [9,10]. It can be helpful in facilitating communication between patients and medical staff, selecting the appropriate therapy, monitoring the results of treatment, and measuring patient-reported outcomes [9,10]. It enables professionals to gain a more complete picture of a patient’s health beyond traditional anatomical and biological indicators. In the future, these questionnaires are likely to become more individualized, with the possibility of being adapted to the specific needs of patients, taking into account changing social and technological realities. Measuring OHRQoL during clinical trials allows the effectiveness of various methods, both therapeutic and preventive, to be assessed. In epidemiological studies, OHRQoL questionnaires help to assess the oral health status of populations, identifying areas that require greater preventive intervention, such as health education or the availability of dental services. In addition, studies on oral-related quality of life have become a key component of analyses of the impact of oral health on other health domains, such as mental health, overall quality of life, or treatment outcomes for other conditions [11]. Integrating quality of life in health policies allows for the application of a personalized approach to the patient [2,12].

OHRQoL can only be assessed indirectly and several tools are used for this purpose, among others: social indicators, global self-item ratings (e.g., visual analog scale—VAS), and multiple item questionnaires. OHRQoL tools are usually multifaceted, allowing the assessment of both physical (e.g., biting difficulties), emotional (e.g., feeling ashamed), and social (e.g., impact on working life) aspects [2,6,12]. Such tools should be based on a theoretical framework. Examples are as follows: Locker’s conceptual model of health, the bio-psycho-social model, the model proposed by Wilson and Cleary, or its modification applicable in children produced by Sischo and Broder [2,7,12,13]. Each tool should pass a validation process, and in the case of application in another culture, also undergo cross-cultural adaptation. It has to feature validity, relevance and acceptability, reliability, responsiveness to change, and interpretability [3,5,9,12,14]. The method of the conducted survey is a self-administrated questionnaire or data can be obtained during a face-to-face interview or via phone interview [5,15]. Nowadays, the instruments are often in electronic form, which facilitates their distribution and analysis.

In recent years, changes in approaches to healthcare have led to the development of various measures designed for OHRQoL evaluation [3,5,8,12]. For adults, Riva et al. [12] identified 42 original instruments having 74 versions: 40 questionnaires for the general population (generic measures) and 34 tools dedicated for patients with particular clinical problems (condition-specific measures). The examples are the Oral Health Impact Profile (OHIP), Geriatric Oral Health Assessment Index (GOHAI), Oral Impact on Daily Performance (ODIP), European Organisation for Research and Treatment of Cancer Quality of Life Questionnaire—Oral Supplement (EORTC QOQ OH-17), or Chewing Function Questionnaire [8,12]. There are also at least eighteen separate scales used in the pediatric population [16]. It is crucial to tailor these instruments to the specific needs of different patient groups, taking into account their age, health status, and cultural background. The advantages of condition-specific instruments are greater sensitivity and the ability to capture even small outcome differences during treatment [17].

Among measures developed for the assessment of OHRQoL in the adult population, the Oral Health Impact Profile (OHIP) is the most widely used and its psychometrical features have been proven [5,12]. The original OHIP questionnaire developed by Slade and Spencer [18] was one of the first tools to assess the social impact of oral diseases. OHIP-49 is a self-completion questionnaire consisting of 49 items selected by an empirical method involving both patients and experts. The measure refers to the patient’s situation over the past year, but initially no recall period was specified [5]. The respondent gives answers rating the frequency of the impairment according to the five-point Likert response format with the following order: “never” coded as 0, “hardly ever” coded as 1, “occasionally” coded as 2, “often” coded as 3 and “very often” coded as 4. The OHIP score can be calculated with the additive method (ADD) as a sum of numerical values of answers [5]. The total value ranges from 0 to 196 and scores for subscales can also be oral health-related quality of life (the higher the OHIP value, the worse the OHRQoL). Another method used to calculate the OHIP results is a simple count (SC) method in which the score is received by counting the number of items with responses regarding one or more frequencies e.g., “occasionally”, “fairly often” or “very often” [19]. According to Reissman [5], this method has some disadvantages because it does not enable to evaluate an impact’s changes. There are also suggestion that OHIP items should be weighted, however, the procedure is burdensome and not enough evidence has been found to prove the usefulness of this approach [3,5]. Recently, the setting of a reference frame to value a patient’s individual score and a minimal important difference (MID) to assess the minimal instrument score changes which are clinically significant are postulated [5].

The theoretical framework of OHIP-49 is based on Locker’s conceptual model of oral health [18]. Locker introduced the concept of oral health-related quality of life, derived from the WHO model of health, indicating that oral conditions can impact an individual’s quality of life [2,20]. Based on his concept, dentists should change their approach from one focused on disease to one focused on the patient [21]. According to Locker, oral disorders can cause impairment, functional limitations, pain and discomfort, disability and handicap with the subsequent outcome of some problems from others [2]. OHIP items are divided into seven domains (subscales). These include: functional limitation, physical pain, psychological discomfort, physical disability, psychological disability, social disability, handicap. Over the years, another construct of OHRQoL has been proposed with four dimensions including psychological impact, orofacial pain, oral functions and orofacial appearance. It was proved that OHIP effectively measures those attributes [22,23].

OHIP-49 has been widely used, and it has been linguistically and culturally adapted and validated in various populations [3,12,24]. Over the years, the measure was critically appraised and some shortcomings were raised. Due to the large number of questions, OHIP-49 is of limited use in children or people with reduced language skills and cognitive abilities. Moreover, the length of the questionnaire makes the assessment time-consuming (estimated time to complete it is about 17 min) and thus can be burdensome to use [18]. The measure is also sensitive to the item order changes and mode of administration [15,25,26]. The concerns about its content validity were raised. Baker et al. [27] found that OHIP-49 contains items that measure more than one domain, but lacks in the adequate representation of all seven constructs. Therefore, this measure has evolved in two directions—the simplification of the questionnaire by reducing the number of questions and the development of OHIP versions designed to assess particular clinical situations. Versions adapted to specific nationalities were also developed. For example, John et al. supplemented the German version (OHIP-G) with 4 additional questions, creating a questionnaire with 53 items [28]. Some authors have used particular OHIP items to create new questionnaires.

There are several versions of the OHIP scale available in the literature. They have been used to compare the subjective perception of oral health depending on disease [29,30], to measure treatment outcome [31,32,33,34,35,36], or even to assess the influence of legislation changes and service use on perceived oral health [37]. OHIP-14 is one of the most commonly applied tools for research on the oral health-related quality of life (OHRQoL), enabling international comparisons to be made [38]. In the clinical setting, using tools such as the OHIP helps to highlight the patient perspective, especially longer versions designed to capture specific oral impairment [5,39]. However, the interpretation of OHIP results can be challenging due to the variability of versions. The results may also be influenced by the psychometric approach, as well as the selection and validation of the tool in the studied population and the accuracy of cross-cultural adaptation [38,40].

To our knowledge, the literature lacks a comprehensive summary of the available Oral Health Impact Profile versions. Therefore, the purpose of this scoping review was to identify and summarize the Oral Health Impact Profile questionnaires available in the literature.

## 2. Materials and Methods

### 2.1. Methodological Framework

For this study, the Joanna Briggs Institute (JBI) scoping review guidelines were adopted [41,42,43]. The Preferred Reporting Items for Systematic Reviews and Meta-Analyses extension for scoping reviews (PRISMA ScR) checklist were followed to ensure good practice in reporting results [44]. The study protocol was developed and approved by all researchers (Ł.W., K.B., O.G., and J.B.). The following approach was used: definition of the research question, development of inclusion and exclusion criteria, establishment of the search strategy and charting, searching for evidence, selection and extraction of evidence, analysis and presentation of results, summary of results.

The research question was framed as follows, “Which Oral Health Impact Profile (OHIP) versions are available to assess oral-health related quality of life in adults?” The research question and inclusion and exclusion criteria were developed based on the PCC (population, concept, context) model which is a recommended approach for scoping reviews [42]. The population (P) was defined as adults, the Oral Health Impact Profile (OHIP) questionnaire was defined as the concept (C), and the context (C) was the oral health-related quality of life. The population was limited to adults because the OHIP was designed for this group.

### 2.2. Inclusion and Exclusion Criteria

This review included articles published without any time restrictions. Original papers, symposium proceedings, literature reviews and meta-analyses, editorials, letters to the editor, and books presenting the Oral Health Impact Profile versions’ development process were included in the review. The exclusion criterion was a lack of explanation as to which items were used and how the scale was modified, as well as OHIP versions which were adapted to particular nationalities, for example, by adding questions specific to such a population. National versions with specific items may have limited applicability in populations other than those for which they were developed. We restricted records to those which were published in English because the use of OHRQoL tools requires their translation and linguistic adaptation, and therefore tools available in languages other than English are far less applicable.

### 2.3. Information Sources and Search Strategy

The search strategy was developed by two authors (J.B. and Ł.W.) and approved by the other researchers (K.B. and O.G.). The following keywords were used to search for relevant publications: [Oral Health Impact Profile OR OHIP] AND adult. Details of the search strategy are shown in Table 1. Three bibliographic databases were searched: PubMed, Scopus, and Web of Science All databases were searched twice for relevant records by two authors (K.B. and O.G.). An initial search was conducted on 16 August 2024, and a final one on 25–28 May 2025. Records were imported into EndNote (Clarivate Analytics, Philadelphia, PA, USA) for the deduplication process. In addition, lists of references in papers were reviewed, with a particular focus on literature reviews and books concerning oral health-related quality of life. A manual search for grey literature using Google’s search tool completed the process of identifying records.

### 2.4. Study Selection and Extraction of Evidence

Titles and abstracts were checked by two researchers (J.B. and Ł.W.). If a researcher had doubts as to whether an article met the inclusion criteria, the other authors were consulted. The form used for data extraction was created by J.B. and approved by the other researchers. The following data were recorded: name of the tool, authors, year of publication, target group, number of questions, language of the tool, OHIP version used to derive a new instrument, items used, period to which the items referred, division into subscales, modifications to the items and to the scale, and validation process. Records were charted separately for generic (i.e., the OHIP version that was not targeted at a specific condition) and condition-specific versions. The analysis of articles qualified for the scoping review was performed independently by two authors (K.B. and O.G.), and the data obtained were reviewed by a third researcher (J.B.). In case of differences, a consensus was reached by discussing the case across the research team. The data extraction form and the accuracy of both researchers involved in data extraction was tested on three studies.

## 3. Results

**Figure 1:**
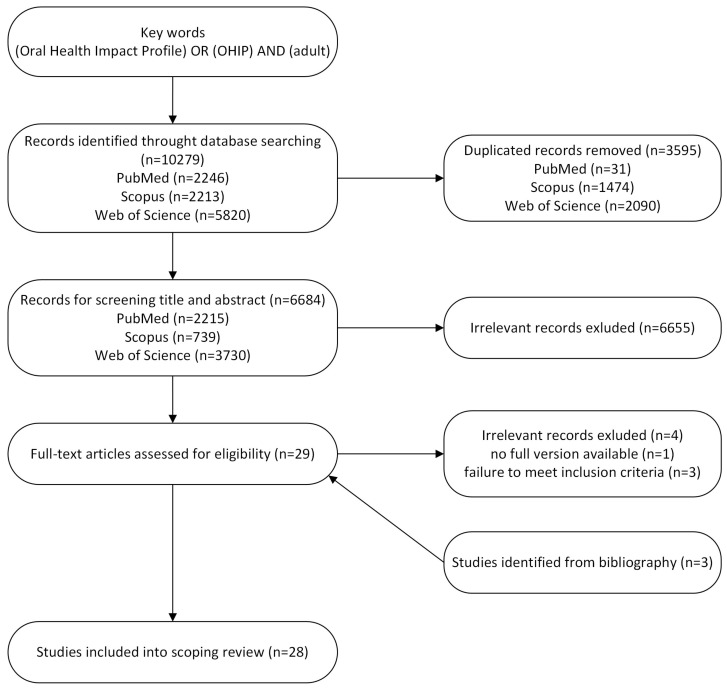
Flow chart.

The results of the literature search gave a total sum of 10,279 records: PubMed—2246; Scopus—2213; and Web of Science—5820. After deduplication, 6684 records were included for title and abstract review. On this basis, 29 records were found for full-text verification. After checking for availability of the full version, one paper was excluded. Three papers were excluded because they did not meet the inclusion criteria. Another three papers were found by searching the list of references in literature reviews, but a search of the grey literature did not reveal any other records. Figure 1 shows the process of acquisition of records.

The final number of records included in this scoping review was 28 (Table 2). It included 11 records of generic OHIP versions dedicated for adults [6,14,18,19,26,27,45,46,47,48] and 16 condition-specified OHIP variants [11,17,32,48,49,50,51,52,53,54,55,56,57,58,59,60,61,62]. Two records were assigned to OHIP-21G [14,45] and OSHIP-Perio [61,62]. Condition-specific OHIPs were designed for patients with periodontal disease [5,55,61,62] or periodontal disease and mucosa lesions [57], patients with temporomandibular disorders [17,49], patients with dental aesthetic problems [11,51], assessment of perception of masticatory efficiency [53], people with mild intellectually deficiency [56], hearing-impaired people [58], patient using fixed orthodontic appliances [59], pregnant women [60], and patients with chronic salivary disorders [32]. There were also two records included in the analysis dedicated for edentulous patients, OHIP-EDENT [50] and POST-OHIP-13 [52], developed for the assessment of treatment outcomes.

### 3.1. Generic OHIPs

Table 3 shows a summary of the features of generic OHIP versions included into this scoping review. The original OHIP-49 and its shortened version OHIP-14 were developed in Australia [6,18]. Other OHIPs designed for the general population originated from Canada [19,27,46], Germany [14,45], the USA [47], Chile [48], and the Netherlands [26]. Tools have been developed continuously since the publication of OHIP-49, whereas the latest paper presenting a general OHIP version was published in 2025 [26].

The original OHIP consisted of 49 items [18]. The abbreviated versions developed for the general population consisted of 46 [14], 42 [46], 22 [27], 21 [14,45], 14 [6,19], 11 [26], 7 [47,48], and 5 [45] items. OHIP-7 and OHIP-5 are also named as ultrashort OHIPs [45,48]. There were a few different recall periods about which patients were asked. Four generic OHIP scales—OHIP-49, OHIP-14, HNANES-OHIP and OHIP-7—referred to 1 year [6,18,47,48], while Locker’s version of OHIP-14 and OHIP-22 [19,27] referred to a period of 3 months (data from Ontario Study of the Oral Health of Older Adults). In the German OHIP-46 and OHIP-21G and in OHIP-42, the recall period was 1 month [14,46]. In the ultrashort German OHIP-5G tool and OHIP *oral discomfort scale,* no recall period was specified [26,45]. Ranges of answers were based on a Likert-type scale with the most often used codes as follows: 4—very often, 3—fairly often, 2—occasionally, 1—hardly ever, and 0—never, with certain scales including an “I don’t know” answer [6,14,18,19,45,48]. In OHIP-22 [27], answers were coded from 5 to 1 with the same gradation of frequency, and NHANES-OHIP used reverse gradation: 1—very often; 5—never [47]. Awad et al. in OHIP-42 [46] implemented more options coded from 6 to 1 (6—all of the time, 5—very often, 4—often, 3—occasionally, 2—rarely, 1—never). Buunk-Werkhoven et al. [26] changed the wording of two responses in their *oral discomfort* scale: 1, “sometimes”; 2, ”regularly” in comparison to the typically used “hardly ever” and “occasionally”.

Among generic OHIP instruments, new versions were derived from OHIP-49 [6,19,27,48], from OHIP-14 according to Slade [26,46,47], and from German instruments developed as the modified versions of German OHIP-53 [14,45]. In general, the authors based their items on the wording of the OHIP-49 questions, but the items used in NHANES-OHIP were reworded [47]. As regards the development procedure, OHIP-49 was designed by experts who chose appropriate statements given by patients during interviews and added items from existing tools [18]. Various approaches were used to shorten OHIP-49: statistical methods were used in seven tools [6,14,26,27,45,48], an expert-based approach in OHIP-46 [14], the item-impact method in OHIP-14 according to Locker and Allen [19], and a combination of methods in OHIP-42 [46]. The method of selection of items for NHANES-OHIP was not specified [47].

Items of OHIP-49 were categorized by seven subscales (domains) of health: functional limitation, physical pain, psychological discomfort, physical disability, psychological disability, social disability, and handicap; OHIP-42 and OHIP-14 both replicated this approach [6,18,19,46]. However, certain tools redefined the dimensional model. John et al. [14] found that only four factors—psychological impact, orofacial pain, oral functions, and appearance—were covered by OHIP-49, and they adapted those subscales in their OHIP-46, OHIP-21G, and OHIP-5G [14,45]. OHIP-22 was found to have six factors with only one subscale referring to the psychological impact [27]. Also, in NHANES-OHIP six subscales had been distinguished due to the combination of psychological discomfort and psychological disability [47]. Buunk-Werkhoven et al. [26] constructed their scale as an instrument assessing oral discomfort, divided into two subscales: psychological and physical discomfort. Almost all generic scales included into this scoping review were validated and assessed for reliability by their authors [6,18,19,24,27,45,46,47]. Different combinations of content, construct, discriminative, concurrent, convergent, and divergent validity tests were conducted. New OHIP versions were also evaluated for sensitivity to change [19], responsiveness [45], internal consistency, and test–retest reliability [46]. In some records, validation was not revealed [14,26,48].

### 3.2. Condition-Specific OHIPs

Table 4 presents results obtained for condition-specific OHIP versions. They were designed by researchers from the Netherlands [49], Canada/UK [50], China [11,55], Denmark [51], UK/Ireland [17], Spain [52], Canada [53], Mexico [54], Portugal [56], Poland [57], India [58], Columbia [59], USA [32,60], and Singapore [61,62]. The first condition-specific OHIP designed for patients with temporomandibular problems [49] was published in 1996, and five scales have been developed during the last five years [32,58,59,60,61,62]. The number of items varies as follows: 30 [49], 22 [17], 19 [50], 18 [55], 14 [11,32,54,56,59,61,62], 13 [52], 12 [58], 7 [53], and 6 [17]. With regard to condition-specific tools, patients were asked about their perceptions over 1 year [56], 1 month [17,49,53,59], and 6 [61,62] and 2 weeks [11]. Two scales, POST-OHIP-13 and the Danish version of OHIP aesthetic, were designed for the assessment of treatment outcomes [51,52]. However, in seven records the time interval was not specified [32,50,54,55,57,58,60]. In most measures, responses were coded from 4 (very often) to 0 (never) [11,17,32,49,50,51,53,55,56,57,58,60,61,62]; in one tool, the answers were coded as always, often, occasionally, rarely, and never [53]. Some authors changed the mode of possible answers for 5-1 [59]. Answers in the POST-OHIP-13 instrument had three options: better (1), the same (0), and worse (−1) [52]. For OHIP-14-PD, the response scale format was not revealed [54].

Taking into consideration condition-specific tools, nine of them were based on OHIP-49 [11,17,49,50,51,55,59,61]. The other six instruments were based on OHIP-14 developed by Slade [32,54,56,57,58,60], and OHIP-PD originated from both instruments [54]. POST-OHIP-13 was derived from OHIP-20 [52]. In five scales, OHIP-30-TMD, OHIP-TMDs, POST-OHIP-13, OHIP-PD, and OSHIP, the authors supplemented the questionnaire with new items appropriate for the condition for which the OHIP version was designed [17,49,52,55,61]. Another common modification introduced to items was a change in wording to reflect the assessed problem [17,32,49,54,56,58,59]. The tool named mOHIP-14 was modified by asking patients the same question separately for teeth, oral mucosa, and dentures [57]. The OHIP version for hearing-impaired people was prepared in a video format [58]. With regard to determining the content of the condition-specific OHIPs, the most common method was based on the opinions of experts or authors [11,49,51,52,53,54,58]. OHIP-s14 Ortho and OSHIP were developed by a statistical approach [59,61], OHIP-EDENT by the item-impact method [50], and OHIP-TMDs and OHIP-CP by the combination of a few methods [17,55]. Five scales were just adaptations of OHIP-14 to specific conditions by changing the wording of items [4,32,56,57,60].

Regarding dimensionality, both tools for temporomandibular disorders, OHIP-EDENT, OHIP aesthetic, OHIP-14-PD, OHIP-14-MID-PT, OHIP-12, OHIP-S14 Ortho, and S-OHIP are distinct in all seven domains of health [5,11,17,32,49,50,54,58,59]. In OHIP-CP, items were distributed between three factors: pain and functional limitation, psychological discomfort and psychological disability, and social handicap [55]. Wąsacz et al. [57] distinguished three factors—psychological and social limitations, physical limitations, and functional limitations—but they assigned different numbers of factors to subscales referring to the particular part of the oral cavity. OSHIP-Perio determined seven domains but only four dimensions and it proposed a new subscale referring directly to periodontal problems [61,62]. Also, MOHIP-14^PW^, even though it consists of the same items as OHIP-14, distinguishes three dimensions—physical impact, psychological impact, and pain impact—instead of seven [60]. In some papers, the dimensionality of the scale was not presented [53]. Similarly to generic variants, measures developed for certain clinical situations were usually validated by the authors and different approaches were used. Lack of validation was found for OHIP aesthetic, OHIP TMD_s_, POST-OHIP-13, OHIP-PD, and S-OHIP [17,32,51,52,54].

## 4. Discussion

The assessment of dPRO has become an essential part of dental care, and the Oral Health Impact Profile measure as one of the most widely used tools for this purpose. Considering the different versions, the OHIP instrument has a very wide range of applications. It is worth noting that the latest versions of the tool were developed in 2024–2025, which justifies our decision to systematize the OHIP versions. There is a general trend toward using short, easy-to-perform, and condition-specific tools and it seems that OHIP-14 is currently the most commonly used version [5,12]. However, short and ultrashort versions of Oral Health Impact Profile consisting of seven and five items have also been developed and validated [5,12]. Some authors recommended OHIP-5 as the most appropriate and indicated that there was no need to develop abbreviated tools [4,5,39]. John et al. [45] found that OHIP-5G captured about 90% of OHIP-49 information. On the other hand, there are reports that OHIP-49 and OHIP-14 perform better than OHIP-5 [63]. There are concerns that reducing the number of items may lead to a loss of content accuracy and the omission of issues important to individual patients [3,45]. The so-called floor phenomenon is highly probable [19]. Awad et al. [46] suggested that reducing the number of items by more than 50% may affect accuracy and that it was best for each domain to be represented by at least two items. Short and ultrashort measures are quicker to administer and may therefore be more useful in clinics and in large surveys. However, before using any shortened version of the OHIP questionnaire its validity and reliability should be carefully assessed to ensure accurate measurement of psychometric properties in the target population.

The usefulness of developing condition-specific scales is questioned by some experts in the field of OHRQoL, with the main concern being the lack of comparability to other disease-specific instruments and measuring the same construct as tools designed for the general population [5]. However, condition-specific measures may be more beneficial for target populations than general tools because they are able to capture the impact of patients’ particular impairments [4,14,64]. The item impact method is recommended for the construction of the questionnaire as it takes into account the patient’s perception of the importance of the item [45]. Our review also showed that some abbreviated OHIP versions may include new items (Table 4, Modifications column). Some authors also made modifications to the wording of items to adapt the scale to the target population.

Various approaches were employed to construct an abbreviated version of the OHIP, which is in accordance with Muelen et al.’s findings [63]. Methods based on statistics, item impact, and expert opinion were most common and widely applied [19,59,63]. The inclusion of two or more approaches is also possible as the process of the development of a new instrument is usually a multi-stage operation and a combination of an expert-based and statistical approaches is preferable [11]. Our scoping review showed that different ways of selecting OHIP items led to the development of various questionnaires [6,11,19], and several variants may be found for the same condition. OHIP-14 has two different versions in which only two items are found in common: “Have you been a bit irritable with other people because of problems with your teeth, mouth or dentures?” and “Have you felt that life in general was less satisfying because of problems with your teeth, mouth or dentures?” There are also several versions of questionnaires available that are applicable to patients with periodontal disease or with other conditions. Therefore, researchers need to be aware of which version they are using to be able to compare the results of their research with other studies.

Another conclusion from this review is that although the range of possible responses is mostly organized according to the Likert scale, they may differ from one another. In most versions, a scale from 0 to 4 is used, where 0 means “never” and 4 means “very often”, and such a format is considered valid [65]. A scale from 1 to 5 was also used to chart patients’ answers. Authors who added an “all of the time” response marked as 6 argued that using a wider response scale allows the patient to choose from the largest number of response categories [45]. Therefore, this means that researchers must take into account the range of the response scale when comparing their research results with those of others. It must be emphasized that international compatibility is a priority for OHRQoL instruments and identical response options are recommended [5,14]. In some studies, the response nomenclature has been changed as compared to the original OHIP scale [26,53]. This aspect should be taken into account during the linguistic and cultural adaptation of a questionnaire.

Determining the appropriate recall period seems to be an important aspect when creating an OHRQoL tool as it may influence the ability to capture a patient’s relevant impairments which may occur with various frequencies. A wide range of recall periods, from one year to two weeks, was adopted when developing OHIP versions. However, according to Reissman [5], the 7-day recall period is sufficient, similar to measures used for general health quality of life assessment.

When it comes to tools evaluating psychometric properties, it is crucial to ensure that the scale actually measures what it is supposed to measure. Some concerns regarding the construct validity of the original OHIP-49 questionnaire have been raised over the years. Baker et al. [27] tested the within- and between-construct validity of OHIP-49 using data from the Ontario Study of Older Adults. They found that the tool may not adequately measure Locker’s oral health model, and that different dimensions may not be distinguishable (especially psychological discomfort and psychological disability). This may be due to the fact that the Locker model is based on theoretical rather than empirical assumptions, or because of the selection of OHIP-49 items that measure similar aspects, as well as due to the population in which the above assessment was conducted [27]. The shortened versions included in the review differed in their approach to OHIP dimensionality and factor structure, with the number of domains/dimensions/factors ranging from 7 to 2 options. Four dimensions of OHRQoL—oral function, facial–oral pain, facial–oral appearance, and psychosocial impact—have been postulated as the best construct for OHRQoL [14,66]. According to Wąsacz et al. [57], the factor structure may vary depending on the part of the oral cavity (e.g., teeth or oral mucosa), and this aspect should be taken into account, with OHRQoL being analyzed in relation to the specific problems of the patient. Buunk-Werkhoven et al. [26] found that OHIP-14 measured satisfaction with dental treatment rather than OHRQoL, and they divided their scale into psychological and physical discomfort. It should be emphasized that the construct validity of different versions of the OHIP was also assessed by other researchers. Possebon at al. [67] proposed for OHIP-EDENT, a three-dimensional model with subscales for physical, psychological, and social impacts. Other studies also confirm our observations regarding discrepancies in the approach to the OHIP structure. According to Campos et al. [64], OHIP-14 measures OHRQoL in different ways depending on the target population with concerns regarding edentulous patients. They confirmed the results obtained by Santos et al. [68] that the unifactorial model is appropriate for OHIP-14. Also, Naik et al. [69] found during the validation of OHIP-5 in English that this measure was a one-dimensional scale and that the data should be presented as a single total score. 

Our analysis has certain limitations. Restricting the literature search to publications written in English may have resulted in the omission of potential results. Moreover, a few versions of the OHIP instrument available in the literature were not included in this analysis. One example is OHIP-G, with four additional questions intended exclusively for the German population [28]. As was stated by its authors, for international comparison purposes the general format should be used [14]. We also did not include OHIP-5E in the analysis because it is OHIP-5G validated in English [69]. The only difference between the source OHIP-5G and the English version is that, according to Naik et al. [69], OHIP-5 is unidimensional and its results should be presented as a single total score. Another excluded version is OHIP-20, an abbreviated version of OHIP-EDENT. This decision was supported by the lack of availability of any article on the development process. The publications by Allen and Locker [50] and Awad et al. [36], which are cited as references for OHIP-20, did not contain detailed information about how that tool was designed. In addition, the questionnaires found in the literature differed in terms of the item that was added to OHIP-EDENT to create OHIP-20 [35,52]. The Oral Health Impact Profile (OHIP) version for young and middle-aged people developed in Japan was omitted due to the lack of access to the full version of the article [70]. We also excluded the OHIP-5_school_ tool [70] because the PPC model established for this scoping review limited the population to adults only. In OHIP-5_school_, the modifications of the original scale were as follows: replacing the word “dentures” with “orthodontic braces” and rephrasing the question about “difficulties in performing daily activities” to a question about “daily activities.” The reference period was limited to the last week [71]. Some of the data included in the analysis did not contain all of the information specified in the study protocol (for example, recall period or range of scale), which also limits this review. The format of data synthesis, a scoping review, did not allow us to compare the quality of data provided by different versions of the OHIP. However, it should be remembered that tools developed for the assessment of psychometric properties should be validated before use, as they may work differently depending on the target group [64].

### Future Research Recommendations

The results of this scoping review revealed the lack of comparability between OHIP versions. This finding is consistent with the recommendations of Jonh et al. [39] that OHIP versions require standardization of features such as the response options or recall period. Future research should also focus on determining the usefulness of short and ultrashort OHIPs in different populations (general and with different problems). Verrips et al. [38] pointed out the need of validation research. Determining the minimal important difference (MID) for different OHIP versions may be helpful in the clinical decision-making process [72]. An important area of research is to define the attributes measured by the OHIP, as various dimensions and factors have been proposed to date.

## 5. Conclusions

This scoping review showed that several versions of the OHIP measure have been developed for both the general population and the specific conditions. These tools differ in many ways, from the number and wording of items, to the range of responses and the period to which the scale refers, to their dimensionality. All of the above-mentioned differences should be taken into account during a comparison of results obtained by different OHIP versions. Due to the availability of various tools, the idea of creating new OHIP versions should be regarded with caution and the use of existing tools should be considered. Moreover, since the process of preparing a tool in a new language and culture is time-consuming and expensive, researchers should select the appropriate OHIP version after careful analysis.

## Figures and Tables

**Table 1:** Search strategy.

Database (Date)	Search Strategy	No. of Records	No. of Records After Deduplication
PubMed (25 May 2025)	(Oral Health Impact Profile [All fields]) OR (OHIP [All fields]) AND (Adult [All fields) AND (adult [filters]) AND (English [filters]))	2246	2215
Web of Science (28 May 2025)	((Oral Health Impact Profile [All Fields]) OR (OHIP [All Fields]) AND (Adult [All Fields]) AND (English [filters])	2213	739
Scopus (28 May 2025)	ALL FIELDS (“Oral Health Impact Profile”) OR ALL FIELDS (“OHIP”) AND ALL FIELDS (“Adult”) AND English (filters) AND adult (filters)	5820	3730

**Table 2:** List of papers included in the scoping review.

OHIP Version	Authors	Year	Population	Country
OHIP-49	Slade GD, Spencer AJ. [19]	1994	general population	Australia
OHIP-14	Slade GD. [6]	1997	general population	Australia
OHIP-14	Locker D, Allen PF. [20]	2002	general population	Canada
OHIP-46	John MT et al. [15]	2004	general population	Germany
OHIP-21G	John MT et al. [15,46]	2004	general population	Germany
OHIP-5G	John MT et al. [46]	2006	general population	Germany
OHIP-22	Baker SR et al. [28]	2008	general population	Canada
OHIP-42	Awad M et al. [47]	2008	general population	Canada
NHANES-OHIP	Sanders AE et al. [48]	2009	general population	USA
OHIP-7	León S et al. [49]	2017	general population	Chile
OHIP *Oral discomfort*	Buunk-Werkhoven YAB et al. [27]	2025	general population	Netherlands
OHIP-30-TMD	Murray H et al. [50]	1996	patients with temporomandibular disorders	Netherlands
OHIP-EDENT	Allen F et al. [51]	2002	edentulous patients	Canada, UK
OHIP- aesthetic	Wong AH et al. [11]	2007	patients with dental aesthetic problems (e.g., teeth discoloration)	China
OHIP *aesthetic outcomes*	Dueled E et al. [52]	2009	evaluation of aesthetic treatment	Denmark
OHIP-TMDs	Durham J et al. [53]	2011	patients with temporomandibular disorders	UK/Ireland
POST-OHIP-13	Montero J et al. [54]	2012	edentulous patients’ treatment outcome	Spain
OHIP *perception of masticatory efficiency*	Cusson V et al. [55]	2015	masticatory efficiency evaluation	Canada
OHIP-PD	Rodríguez NI et al. [56]	2017	patients with periodontal disease	Mexico
OHIP-CP	He S et al. [57]	2017	patients with chronic periodontal disease	China
OHIP-14-MID-PT	Couto P et al. [58]	2018	adults with mild intellectual disabilities	Portugal
mOHIP-14	Wąsacz K et al. [59]	2019	patients with mucosa lesions or periodontal disease	Poland
OHIP-12 *sign lang.*	Sulekha SG et al. [60]	2020	hearing-impaired people	India sign language
OHIP-s14-Ortho	Barrera-Chaparro JP et al. [61]	2021	adults and children with fixed orthodontic appliances	Columbia
MOHIO-14^PW^	Yang C et al. [62]	2022	pregnant women	USA
S-OHIP	Coca KK et al. et al. [63]	2023	patients with chronic salivary disorders	USA
OSHIP-Perio	Wong LB et al. [64,65]	2024	patients with periodontal disease	Singapore

**Table 3:** Summary of generic OHIP versions.

Name	Items	Recall Period	Range of Responses	Development/ Item Selection Process	Subscales (No. of Items According to OHIP-49)	Modifications	Reliability/ Validation
OHIP-49 [18]	49	1 year	4—very often 3—fairly often 2—occasionally 1—hardly ever 0—never I don-t know	535 statements received during interviews with patients were assessed by experts who chose 46 items, and 3 additional items were added from existing tools items relocated to seven domains based on Locker’s conceptual model	7 domains functional limitation (1–9), physical pain (10–18), psychological discomfort (19–23), physical disability (24–32), psychological disability (33–38), social disability (39–43), handicap (44–49)		tested for convergent validity
OHIP-14 [6]	14	1 year	4—very often 3—fairly often 2—occasionally 1—hardly ever 0—never I don-t know	derived from OHIP-49 by eliminating 3 questions regarding denture-related problems and 7 questions where 5% or more of responses were left unanswered or answered “I don’t know” statistical approach (internal reliability, principal component factor, and least-squares regression) used to choose final set of items (two items per dimension)	7 dimensions functional limitation (2, 6), physical pain (9, 15), psychological discomfort (20, 23), physical disability (29, 32), psychological disability (35, 38), social disability (42, 43), handicap (47, 48)		tested for discriminative validity
OHIP-14 [19]	14	3 months	4—very often 3—fairly often 2—occasionally 1—hardly ever 0—never I don-t know	derived from OHIP-49 by the item-impact method two top-scoring items from each subscale	7 subscales: functional limitation (1, 7), physical pain (12, 16), psychological discomfort (19, 21), physical disability (24, 28), psychological disability (34, 36), social disability (40, 42), handicap (45, 47)		tested for reliability, content, discriminative and concurrent validity, sensitivity to change, and stability
OHIP-46 [14]	46	1 month	4—very often 3—fairly often 2—occasionally 1—hardly ever 0—never	derived from OHIP-53G (German version of OHIP-49) by exclusion of 4 items specified for German population and 3 items Q9, Q18, and Q30 referring to dentures	4 factors psychological impact, orofacial pain, oral functions, appearance	re-framework of the dimensional structure by the exploratory factor analysis (4 factors) indicated in younger populations	
OHIP-21 [14,45]	21	1 month	4—very often 3—fairly often 2—occasionally 1—hardly ever 0—never	based on OHIP-G and two versions of OHIP-14 items selected by the factor of analytic method used in other study (John Dimensions)	4 dimensions psychological impact (36, 37, 38, 40, 42, 43, 48, 49), orofacial pain (10, 11, 13, 14, 15, 17), oral functions (1, 2, 4), appearance (3, 19, 22)	items loaded to 4 dimensions	tested for content and construct validity, responsiveness, and reliability
OHIP-5 [45]	5	not stated	4—very often 3—fairly often 2—occasionally 1—hardly ever 0—never	based on OHIP-G and two versions of OHIP-14 regression models with clinical impact	4 dimensions psychological impact (43), orofacial pain (10), oral functions (1), appearance (22) item without dimension: 26	items loaded to 4 dimensions	tested for content and construct validity, responsiveness, and reliability
OHIP-22 [27]	22	3 months	5—very often 4—fairly often 3—occasionally 2—hardly ever 1—never I don-t know	derived from OHIP-49 items selected by confirmatory factor analysis	6 factors functional limitation (1, 2, 3, 4, 6, 7), pain (9, 11, 16), psychological impact (21, 23, 34, 35, 36), physical disability (27, 28, 29, 32), social disability (40, 41), handicap (45, 48)	scale re-specified to the six-factor model	tested for within- and between-construct validity
OHIP-42 [46]	42	1 month	6—all of the time 5—most of the time 4—some of the time 3—occasionally 2—rarely 1—never I don-t know	derived from OHIP-14 by the expert panel and item-impact method and mean frequency rating	7 domains functional limitation (1, 2, 4–9), physical pain (10, 12, 15, 17, 18), psychological discomfort (19, 20, 22–23), physical disability (24–26, 28–32), psychological disability (33–38), social disability (39–43), handicap (44–49)	range of possible answers wider than in OHIP-49	tested for reliability and construct validity
NHANES-OHIP [47]	7	1 year	1- very often 2—fairly often 3—occasionally 4—hardly ever 5—never I don’t know	7 items derived from OHIP-14 according to Slade	6 dimensions functional limitation (6), physical pain (10, 16), psychological discomfort combine with psychological disability (20 combined with 38), physical disability (28), social disability (43), handicap (47)	some wording changes compared to OHIP-14 the reverse codes were used	tested for construct validity and adequacy
OHIP-7 [48]	7	1 year	4—very often 3—fairly often 2—occasionally 1—hardly ever 0—never	derived from OHIP-49 one item from each dimension was selected by linear regression	7 dimensions functional limitation (8), physical pain (13), psychological discomfort (21), physical disability (24), psychological disability (33), social disability (43), handicap (48)		
OHIP *Oral discomfort* [26]	11	not stated	4—very often 3—fairly often 2—regularly 1—sometimes 0—never	derived from OHIP-14 by confirmatory factor analysis and simultaneous components analysis	2 factors psychological discomfort (20, 42, 38, 23, 47, 35), physical discomfort (10, 43, 16, 32, 29)	one-factor scale for assessment of oral discomfort with two subscales from dimensions of psychological discomfort and physical discomfort change in most often used options of answers coded 1 and 2	

**Table 4:** Summary of condition-specific OHIP versions.

Name	Items	Recall Period	Range of Responses	Development/ Item Selection Procedure	Subscales (No. of Items According to OHIP-49)	Modifications	Reliability/ Validation
OHIP-30-TMD [49]	30	1 month	4—very often 3—fairly often 2—occasionally 1—hardly ever 0—never	panel of experts reduced items from OHIP-49 (physical pain items and those not relevant to the facial pain were removed)	7 subscales functional limitation (1, 2, new item), psychological discomfort (19–21, 23), physical disability (24, 25, 27, 28, 43, 32, 16), psychological disability (33–38), social disability (39–42, new item, 31), handicap (46–49)	the expression “teeth, mouth, or dentures” changed into “pain” new items added: “taking longer to complete meals” and “avoiding eating with others” some items relocated to the other domain	tested for internal consistency
OHIP-EDENT [50]	19	not stated	4—very often 3—fairly often 2—occasionally 1—hardly ever 0—never	derived from OHIP-49 item impact method used in two populations top two ranked items from each domain from each population (Australian and British) were included	7 domains functional limitation (1, 7, 9), physical pain (10, 16, 17, 18), psychological discomfort (19, 20), physical disability (28, 32, 30), psychological disability (34, 38), social disability (40, 42, 39), handicap (46, 47)		tested for sensitivity to change and stability
OHIP- aesthetic [11]	14	2 weeks	4—very often 3—fairly often 2—occasionally 1—hardly ever 0—never	derived from OHIP-49 two approaches were tested: the conceptual method based on the experts choice of the most suitable items vs. the regression methods the expert-based approach showed better sensitivity for assessing dental aesthetics outcomes	7 domains functional limitation (3, 4), physical pain (13, 17), psychological discomfort (20, 22), physical disability (26, 31), psychological disability (35, Q8), social disability (40, 43), handicap (46, 47)		tested for internal reliability, discriminating ability, responsiveness, and sensitivity to change
OHIP *aesthetic outcome* [51]	6	after treatment	4—very often 3—fairly often 2—occasionally 1—hardly ever 0—never	derived from OHIP-49 items selected by authors	no subscales Items: 3, 4, 20, 22, 31, 38		
OHIP-TMDs [17]	22	1 month	4—very often 3—fairly often 2—occasionally 1—hardly ever 0—never	mixed methods including statistical analysis of OHIP-49 (quantitative study) and interviews (qualitative study) supported by the authors’ experience	7 domains functional limitation (1, new item), physical pain (10, 11, 12, 16, new item), psychological discomfort (19, 20, 21, 23), physical disability (28, 32), psychological disability (33, 34, 35, 36, 37), social disability (42, 43), handicap (47, 49)	OHIP 49 items were modified by adding “jaws” to the expression “teeth, mouth, or dentures” two new items derived from qualitative study: “Have you had difficulties in opening and closing your mouth?” and “Have you felt speech was painful because of problems with your teeth, mouth, dentures, or jaws?”	
POST-OHIP-13 [52]	13	after treatment	1—better 0—the same −1—worse	derived from OHIP-20 by the authors’ expertise for the assessment of the OHRQoL after prosthodontic treatment items: 1: chewing, 2: food packing, 3: satisfaction with diet, 4: pain/discomfort, 5: presence of ulcers, 6: denture fits, 7: denture retention, 8: denture comfort, 9: smile, 10: teeth shape, 11: color and position, 12: oral well-being, 13: satisfaction with life	not specified	new items regarding smile, artificial teeth shape, color, and position of artificial teeth	
OHIP *perception of masticatory efficiency* [53]	7	4 weeks	4—always 3—often 2—occasionally 1—rarely 0—never	derived from OHIP-49 by authors items measuring perception of masticatory efficiency chosen by authors (no. 1, 28, 32, 30, 16, 9, 18)	not specified		tested for internal consistency
OHIP-PD [54]	14	not stated	not stated	derived from OHIP-14 (Slade) and OHIP-49 by expert panel	7 dimensions: functional limitation (3 modified, 1 modified), physical pain (15, 13), psychological discomfort (19 modified, 22 modified), physical disability (27 modified, 28 modified), psychological disability (34 modified, 38 modified), social disability (42 modified, 49 modified), handicap (44 modified, 45 modified)	items adapted to periodontal disease by modifying the wording	
OHIP-CP [55]	18	not stated	4—very often 3—fairly often 2—occasionally 1—hardly ever 0—never I don-t know	phase I: derived from OHIP-49 and other literature sources by panel of experts phase II: quantitative classical test theory and qualitative content analysis with second panel of experts, investigators, and patients phase III: validation	3 factors pain and functional limitation (14, 15, 13, new item, 1, new item, 5, 7), psychological discomfort (6, 20, 23, 39, 27, 29), psychological disability and social handicap (31, 38, 44, 47)	items relocated into 3 domains New items: “Have you ever had any teeth become loose on their own, without any injury?”, “Have you had bleeding gums spontaneously bleeding or while brushing your teeth or biting hard objects?”	tested for construct, discriminative, and convergent validity, internal consistency, and test–retest reliability
OHIP-14-MID-PT [56]	14	1 year	4—very often 3—fairly often 2—occasionally 1—hardly ever 0—never	OHIP-14 Slade’s version adapted to mild intellectually disable people by collecting data during interview instead of by the self-administrative questionnaire	7 dimensions functional limitation (2, 6), physical pain (9, 15), psychological discomfort (20, 23), physical disability (29, 32), psychological disability (35, 38), social disability (42, 43), handicap (47, 48)	several modifications to the wording of items	tested for construct, convergent, and divergent validity, internal consistency, and test–retest reliability
mOHIP-14 [57]	14	not stated	4—very often 3- fairly often 2- occasionally 1—hardly ever 0—never 5—I don’t know 6—not applicable	based on OHIP-14 Slade’s version	Each subscale has a different number of factors in the model: Subscale 1—three-factor model psychological and social limitations (23, 38, 20, 42, 47, 48, 43), physical limitations (9, 32, 35, 15, 29), functional limitations (2, 6) Subscale 2—single-factor model Subscale 3—two-factor model social interaction limitations (20, 15, 38, 23, 9, 32, 29, 35), basic activities disorder and personal discomfort (48, 43, 47, 42, 2, 6)	three subscales obtained by asking the same question in relation to teeth (subscale 1), oral mucosa (subscale 2), and dentures (subscale 3) additional answer “not applicable” for toothless patients in subscale 1 or patients not using dentures in subscale 2 variation in the factors structure depending on the part of the oral cavity	tested for internal consistency
OHIP-12 *Sign lang.* [58]	12	not stated	4—very often 3—fairly often 2—occasionally 1—hardly ever 0—never	derived from OHIP-14 by expert panel questionnaire in sign language in video format two items removed: “Have you had trouble pronouncing any words?” and “Have you been totally unable to function?”	7 domains functional limitation (1 question), physical pain (2 questions), psychological discomfort (5 questions), physical disability (1 question), psychological disability (1 question), social disability (1 question), handicap (1 question)	items relocated within domains some changes made to the wording due to difficulties in understanding the signs for words such as “shy,” “embarrassed,” and “irritable.”	tested for internal consistency and test–retest reliability
OHIP-S14 Ortho [59]	14	1 month	5—very often 4—fairly often 3—occasionally 2—hardly ever 1—never	derived from OHIP-49 by exploratory factor analysis and confirmatory factor analysis	7 dimensions functional limitation (1, 4), physical pain (10, 15), psychological discomfort (22, 23), physical disability (31, 32), psychological disability (34, 35), social disability (42, 43), handicap (47, 48)	the expression “teeth, mouth, or dentures” changed into “brackets” rephrasing some items	tested for content and construct validity, internal consistency, and test–retest reliability
MOHIP-14^PW^ [60]	14	not stated	4—very often 3—fairly often 2—occasionally 1—hardly ever 0—never	derived from OHIP-14	3 dimensions physical impact (2, 6, 29, 32, 35, 42, 43, 47, 48), psychological impact (20, 23, 38), pain impact (9, 15)	reducing the number of dimensions from 7 to 3 items’ redistribution within subscales based on confirmatory and exploratory factor analysis	tested for internal consistency and test–retest reliability
S-OHIP (14) [32]	14	not stated	4—very often 3—fairly often 2—occasionally 1—hardly ever 0—never	derived from OHIP-14	7 domains functional limitation (1, 4), physical pain (10, 15), psychological discomfort (22, 23), physical disability (31, 32), psychological disability (34, 35), social disability (42, 43), handicap (47, 48)	OHIP-14 (Slade) modified by changing the expression “teeth, mouth, or dentures” into “salivary problem”	
OSHIP-Perio [61,62]	14	6 weeks	4—very often 3—fairly often 2—occasionally 1—hardly ever 0—never	preliminary OSHIP-Perio derived from OHIP-49 and additional 8 items specific to the periodontal disease added by the expert panel based on a previous study probability-based model used to reduce items of preliminary OSHIP-Perio additional items specific to periodontitis	7 domains periodontal: 5 new items, functional limitations (1, 2, 8), physical pain (13, 14, 15, 16, physical disability (26), handicap (44) 4 dimensions periodontal: 5 new items, oral function (1, 2), orofacial pain (13, 14, 15), oral function (16, 26), psychological impact (44), item without dimension (8)	exploratory factor analysis used to evaluate dimensionality of preliminary OSHIP-Perio (Oral and Systematic Health Impact Profile) four-dimensional model with a new dimension: periodontal New items: “Have you experienced shaky teeth?”, “Have you experienced bleeding gums?”, “Have you experienced abnormal taste in your saliva?”, “Have you felt that your gums affect your underlying medical conditions/medications affect your gums?”, “Have you felt that your gums affect your underlying medial conditions?”	tested for discriminant, convergent, and concurrent validity, internal consistency, and test–retest reliability

## Data Availability

The original contributions presented in this study are included in the article. Further inquiries can be directed to the corresponding author.

## Abbreviations

The following abbreviations are used in this manuscript:

WHO	World Health Organization
OHRQoL	Oral health-related quality of life
dPRO	Dental patient-related outcomes
VAS	Visual analog scale
OHIP	Oral Health Impact Profile
GOHAI	Geriatric Oral Health Assessment Index
ODIP	Oral Impact on Daily Performance
EORTC QLQ OH-17	European Organisation for Research and Treatment of Cancer Quality of Life Questionnaire. Oral Supplement
ADD	Additive method
SC	Simple count method
MID	Minimal important difference
JBI	Joanna Briggs Institute
PRISMA Scr	The Preferred Reporting Items for Systematic Reviews and Meta-Analyses extension for scoping reviews
PCC	Population, concept, context
*sign lang*	Sign language
